# Serum Amyloid A1/Toll-Like Receptor-4 Axis, an Important Link between Inflammation and Outcome of TBI Patients

**DOI:** 10.3390/biomedicines9060599

**Published:** 2021-05-25

**Authors:** Víctor Farré-Alins, Alejandra Palomino-Antolín, Paloma Narros-Fernández, Ana Belen Lopez-Rodriguez, Céline Decouty-Perez, Alicia Muñoz-Montero, Jorge Zamorano-Fernández, Beatriz Mansilla-Fernández, Javier Giner-García, Pablo García-Feijoo, Miguel Sáez-Alegre, Alexis J. Palpán-Flores, José María Roda-Frade, Cristina S. Carabias, Juliana M. Rosa, Belén Civantos-Martín, Santiago Yus-Teruel, Luis Gandía, Alfonso Lagares, Borja J. Hernández-García, Javier Egea

**Affiliations:** 1 Molecular Neuroinflammation and Neuronal Plasticity Research Laboratory, Research Unit, Hospital Universitario Santa Cristina, Instituto de Investigación Sanitaria-Hospital Universitario de la Princesa, 28009 Madrid, Spain; victorfarre@hotmail.com (V.F.-A.); alejandra.palominoantolin@gmail.com (A.P.-A.); palomanf22@gmail.com (P.N.-F.); lopezrodr.ab@gmail.com (A.B.L.-R.); celinedecouty96@gmail.com (C.D.-P.); juli.martins.rosa@gmail.com (J.M.R.); 2 Instituto Teófilo Hernando, Departamento de Farmacología y Terapéutica, Facultad de Medicina, UAM, 28029 Madrid, Spain; alimunozmontero@gmail.com (A.M.-M.); luis.gandia@uam.es (L.G.); 3 Servicio de Neurocirugía, Hospital Universitario La Paz, 28046 Madrid, Spain; teveosabio@gmail.com (J.Z.-F.); beatrizmf25@gmail.com (B.M.-F.); Javier.giner.garcia@gmail.com (J.G.-G.); g.feijoo.neurosurgery@gmail.com (P.G.-F.); miksaezalegre@gmail.com (M.S.-A.); alexispalpan@hotmail.com (A.J.P.-F.); josem.roda@salud.madrid.org (J.M.R.-F.); borjajhega@gmail.com (B.J.H.-G.); 4 Servicio de Neurocirugía, Hospital Universitario 12 de Octubre, imas12, Universidad Complutense de Madrid, 28041 Madrid, Spain; csanchezcarabias@gmail.com (C.S.C.); Algadoc@yahoo.com (A.L.); 5 Servicio de Medicina Intensiva, Hospital Universitario La Paz, 28046 Madrid, Spain; bcivantos@hotmail.com (B.C.-M.); santiagoyus@gmail.com (S.Y.-T.)

**Keywords:** traumatic brain injury, neuroinflammation, prognosis, biomarkers, immunomodulation

## Abstract

Traumatic brain injury (TBI) is one of the leading causes of mortality and disability worldwide without any validated biomarker or set of biomarkers to help the diagnosis and evaluation of the evolution/prognosis of TBI patients. To achieve this aim, a deeper knowledge of the biochemical and pathophysiological processes triggered after the trauma is essential. Here, we identified the serum amyloid A1 protein-Toll-like receptor 4 (SAA1-TLR4) axis as an important link between inflammation and the outcome of TBI patients. Using serum and mRNA from white blood cells (WBC) of TBI patients, we found a positive correlation between serum SAA1 levels and injury severity, as well as with the 6-month outcome of TBI patients. SAA1 levels also correlate with the presence of TLR4 mRNA in WBC. In vitro, we found that SAA1 contributes to inflammation via TLR4 activation that releases inflammatory cytokines, which in turn increases SAA1 levels, establishing a positive proinflammatory loop. In vivo, post-TBI treatment with the TLR4-antagonist TAK242 reduces SAA1 levels, improves neurobehavioral outcome, and prevents blood–brain barrier disruption. Our data support further evaluation of (i) post-TBI treatment in the presence of TLR4 inhibition for limiting TBI-induced damage and (ii) SAA1-TLR4 as a biomarker of injury progression in TBI patients.

## 1. Introduction

Traumatic brain injury (TBI) is one of the leading causes of mortality and disability worldwide, and it is usually initiated after a blunt impact, penetration through the skull into the brain, or exposure to explosive blast. TBI has been classified from different points of view including the classic severity score according to the degree of alteration of the consciousness level (mild, moderate, or severe), or according to the type of injury identified in the computed tomography (focal or diffuse) [1,2]. TBI has a complex pathophysiology that produces mechanical injury within seconds to neurons, glia, and blood vessels. This primary damage triggers a secondary process from different etiologies, i.e., ischemia, excitotoxicity, edema, or inflammation, that evolves from weeks to months [3,4]. In animals, inflammation is increasingly recognized to be an important cause of secondary brain injury, and it is initiated by the release of damage associated molecular patterns (DAMPs) from necrotic cells leading to the activation of astrocytes and microglia [5]. Release of proinflammatory cytokines and chemokines lead to further breakdown of the Blood–Brain Barrier (BBB) and recruitment of peripheral inflammatory cells [6].

Toll-like receptors (TLRs) are a class of transmembrane pattern-recognition receptor family that play a key role in the activation of innate immune system in response to pathogens (infection), or to resolve damage produced by DAMPs (sterile response) [3,4,5,6,7]. TLR4 is highly expressed in macrophage/microglia and its activation by different DAMPs (i.e., HMGB1 or Hsp90) initiates an inflammatory cascade in several acute CNS-pathologies and after brain injuries [8]. It has been recently shown that the absence of TLR4 protects against TBI by affecting the polarization of microglia, and therefore decreasing the inflammatory response [9]. Moreover, TLR4 inhibition by resatorvid (TAK242) decreases the development of the secondary injury after TBI in a mouse model [10]. Although all the aforementioned strongly support that TLR4 has a crucial role for the pathophysiology of TBI, its contribution to the inflammatory process to secondary TBI in the clinical scenery is less well established.

A biomarker is a surrogate and objective indicator of biological processes occurring in an individual that provides information about the pathology of a disease/condition or the response to a pathogen or to a treatment [11]. In the context of TBI, biomarkers have the potential to be used as diagnostic markers of injury severity, response to treatment (monitoring biomarkers), or even as predictors of outcomes (predictive biomarkers). Noninvasive and feasible techniques such as the use of plasma biomarkers are a priority in current medicine [11,12,13]. Discovering biomarkers that help us make decisions in clinical practice in a patient who has recently suffered a TBI is essential to determine the biochemical and pathophysiological processes that are triggered after the trauma [13]. The Serum Amyloid A1 (SAA1) protein is one of the acute-phase response proteins, mainly synthetized in the liver, which is released into the systemic circulation in response to inflammation [14]. Recently, we have found that the SAA1 protein is a potential intracranial and extracranial clinical severity biomarker in TBI [15]. The aim of this study was to evaluate the individual and combined outcome prediction ability of SAA1 and TLR4, both related with inflammation and to compare them to S100β, a multifunctional protein that is found in astrocytes, used as biomarker TBI [12,13,14]. Moreover, as SAA1 may serve as a DAMP, we hypothesized that SAA1 may be an important link between inflammation and the outcome of TBI patients. To validate this hypothesis, we examined the relation of SAA1 and TLR4, both in samples of TBI patients and in the in vivo model of closed head injury (CHI) in mice, as well as in in vitro cultures of glial cells.

## 2. Materials and Methods

### 2.1. Study Approval

All animal procedures and protocols (PROEX 109/18) were conducted in compliance with the Guide for the Care and Use of Laboratory Animals and approved by the Ethics Committee of Universidad Autónoma de Madrid (Madrid, Spain) and followed the ARRIVE Guidelines. The clinical study protocol was conducted in accordance with the ethical standards of the institutional review board approved by the Ethics Committee of Hospital Universitario La Paz (PI-2153). At admission, informed consent was obtained from all patients (or their relatives in those patients presenting decreased level of consciousness).

### 2.2. Human Samples 

This observational prospective cohort study was conducted at the Department of Neurosurgery of Hospital Universitario La Paz between April 2017 and October 2018. Patients were included in accordance with the following criteria: age between 18 and 85 years, admitted to our hospital with the diagnosis of closed head injury, presenting within 24 h of injury, and admitted to the Intensive Care Unit or Neurosurgery Ward. Exclusion criteria were as follows: presence of previous neurological disease or cognitive impairment or inability to perform head CT, to collect biological sample, or to complete a proper follow-up. Venous blood samples were obtained at 24 h, 72 h, and 7 days after suffering TBI. Blood was allowed to clot, and after centrifugation (1000× *g*, 30 min) serum was stored at −80 °C until analysis. A group of healthy controls, without clinical history of TBI or major disease, were randomly collected with the objective of detecting those proteins present in patients with TBI and comparing biomarker levels with the study cohort.

### 2.3. Intracranial Clinical Severity Evaluation

The impairment of consciousness levels was assessed by the GCS at hospital admission. Mild injury was defined as a GCS score of 14 to 15. Moderate injury was defined as a GCS of 9 to 13, and severe injury was defined as a GCS score of 3 to 8 (regardless of CT scan findings).

### 2.4. Outcome

Outcomes were evaluated in person or by mail or telephone with the patient or a close relative using a validated questionnaire for assessment. Outcomes were ascertained using the 6 months Glasgow Outcome Scale extended (GOSE). For statistical analysis, GOSE was dichotomized into favorable (8/7/6) versus unfavorable (5/4/3/2/1) outcome.

### 2.5. ELISA Assay

Human blood was used to determine the levels of SAA1 with a specific ELISA (Cat. No. EL10015, Anogen, ON, Canada) and the levels of S100B by CLIA (Cat. No. LIAISON S100B 314701, Diasorin, Italy). Blood from mice was collected in EDTA-treated tubes and centrifuged at 2500 rpm for 10 min to obtain serum. Cortex from contralateral and ipsilateral hemispheres of mice brains were lysed in RIPA lysis buffer (0.5% Nonidet P-40, 0.1% sodium dodecyl sulfate, 137 mM NaCl, 2.7 mM KCl, 10 mM Na2HPO4, 2 mM KH2PO4) and 100 µg of protein was used to determine SAA1 levels. Cell culture supernatants from mixed glial cultures were frozen until protein quantification. IL-1β, TNF-α, and SAA1 levels from brains and glial cultures were measured by specific ELISA kits (Cat. No. DY401 for IL-1β, DY410 for TNF-α, DY2948 for SAA1; R&D Systems, Minneapolis, MN, USA) according to manufacturer’s protocols.

### 2.6. Animals

Three-month male C57BL/6J mice (25–30 g were used to perform the experiments. Animals were group-housed under controlled temperature and lighting conditions and ingested water and food ad libitum. Every effort was made to reduce the number of animals used and their suffering. 

### 2.7. Mixed Glial Cultures

Mixed glial cultures were prepared from cerebral cortices of 4-day-old C57BL/6J mice as previously described [16]. Briefly, meninges and blood vessels were removed, and forebrains were dissociated by repeated pipetting in DMEM/F12 medium (Fisher Scientific, Madrid, Spain). Cells were seeded in DMEM/F12 with 20% FBS at a density of 3 × 10^5^ cells/mL and maintained at 37 °C in humidified 5% CO_2_/95% air. Confluence was reached at 10–12 days in vitro and cultures were treated with SAA1 recombinant protein (R&D Systems, Minneapolis, MN, USA), TNF-α recombinant protein (R&D Systems, USA), IL-1β recombinant protein (R&D Systems, USA), LPS (Sigma-Aldrich, Madrid, Spain), and TAK242 (Sigma-Aldrich, Madrid, Spain) in DMEM/F12 with 10% FBS. 

### 2.8. Closed Head Injury (CHI) Model

CHI is a weight drop model in which the skull is exposed to a free-falling weight. Procedures were adapted from Flierl et al. [17]. The injury was produced to the right hemisphere between sagittal and lambdoid sutures by a weight dropped from 34 cm and weighing 50 g in order to produce severe TBI (assessed 1 h after trauma by Neurological Severity Score, described below). The head of the animals was placed on a hard surface to reduce the dissipation of energy and generate a focal injury. Two hours before CHI, animals were treated with buprenorphine 0.05 mg/kg subcutaneously for analgesia. Mice were anesthetized with inhaled isoflurane before the impact and subjected to oxygen administration after trauma until regular breath was restored.

Mice were divided in 3 groups: sham, not subjected to CHI; vehicle, treated with 0.9% NaCl containing 3% DMSO; TAK242, treated with the TLR4 inhibitor TAK242 at 3 mg/kg diluted in 0.9% NaCl containing 3% DMSO. 

### 2.9. Neurological Severity Score (NSS) Test

The assessment of the severity of the injury, which correlates with the weight falling height, is based on the evaluation of motor and neurobehavioral functions at 1 h after trauma using the Neurological Severity Score (NSS), modified from Flierl et al. [17]. The score consists of the evaluation of behavioral and neurological parameters, in which 10 points means a total neurological impairment. Mice with a score of 9 and 10 points were sacrificed to avoid suffering according to the Guide for the Care and Use of Laboratory Animals. Animals were treated intraperitoneally (i.p.) after the 1 h neurological test. NSS were performed again at 24 h to determine possible differences between groups.

### 2.10. Blood–Brain Barrier Integrity Assessment

Evans Blue tracer (Sigma-Aldrich, Madrid, Spain) diluted at 2% in saline was injected i.p. immediately after CHI for 1 h post-TBI analysis, following the 1-h NSS test for 24 h post-TBI analysis, or 6 days post-TBI for 7 days post-TBI analysis. Animals were sacrificed 1 h, 24 h or 7 days after trauma and brains were extracted and sectioned in four 2 mm slices using a mouse brain slicer. The slices were scanned, and total area of Evans Blue extravasation was measured in each slice using the program ImageJ 1.52e (ImageJ software, National Institutes of Health, Stapleton, NY, USA).

### 2.11. RNA Extraction and Quantitative Real-Time PCR

Total RNA from blood cells of controls and trauma patients were obtained using the QIAamp^®^ RNA Blood Mini Kit (Cat No. 52304, Qiagen, Germany). cDNA was synthetized with the iScript cDNA synthesis kit (Cat No. 1708891, Biorad, Hercules, CA, USA) and real-time PCR was performed on a QuantStudio 5 PCR system (Applied Biosystems, Foster City, CA, USA). Expression of human TLR4 was analyzed using Taqman^®^ gene expression assays (FAM probe, Hs00152939_m1). mRNA expression was normalized against eukaryotic 18S rRNA (VIC probe, 4310893E) in the same samples and calculated by the Δ/Δ Ct method.

### 2.12. Immunoblotting and Image Analysis

Mouse brain cortex and glial cultures were lysed in RIPA lysis buffer (0.5% Nonidet P-40, 0.1% sodium dodecyl sulfate, 137 mM NaCl, 2.7 mM KCl, 10 mM Na2HPO4, 2 mM KH2PO4). Thirty micrograms of protein were resolved by SDS–PAGE and transferred to Immobilon-P membranes (Millipore Corp., Billerica, MA, USA). Membranes were incubated with the primary antibodies anti-iNOS (1:1000; Cat No. sc-650, Santa Cruz Biotechnology, Dallas, TX, USA), anti-SAA1/A2 (1:1000; Cat No. AF2948, R&D Systems, USA) or anti-β-actin (1:50,000; Cat No. A3854, Sigma-Aldrich, Madrid, Spain). Appropriate peroxidase-conjugated secondary antibodies (1:5000; Cat Nos. sc-2354 and sc-2357, Santa Cruz Biotechnology, USA) were used to detect proteins by enhanced chemiluminescence. Different band intensities were quantified using the Scion Image program (RRID:SCR_008673). Immunoblot images correspond to a representative experiment.

### 2.13. Immunofluorescence

Mice were anesthetized i.p. with ketamine (100 mg/kg)/xylazine (10 mg/kg) and through vascular perfusion brains were washed with saline solution to remove hemorrhagic areas. Brains were extracted, embedded in OCT media, frozen at −80 °C, and sectioned in 15 µm thick slices using a cryostat (CM 1100, Leica Microsystems, Madrid, Spain). Brain sections were fixed with 4% paraformaldehyde dissolved in PBS for 10 min and washed three times with PBS every 5 min. After 2 h of blocking with 20% goat serum, tissue was incubated overnight at 4 °C with the primary antibodies anti-APP (1:1000; Cat No. 36-6900, ThermoFisher Scientific, Waltham, WA, USA) or anti-SAA1/A2 (1:100; Cat No. AF2948, R&D Systems, USA) diluted in PBS with 0.3% Triton X-100. Then, incubation with fluorescent secondary antibodies (1:500; Cat Nos. A-11057 and A-21206, Invitrogen, Carlsbad. CA, USA) and nuclei staining with DAPI (1:1000; Cat No. D1306, ThermoFisher Scientific, USA) was performed. Finally, the slides were covered with coverslips adding the mounting medium Fluoromount-G (SouthernBiotech, Birmingham, AL, USA). Immunofluorescence images were obtained using a Leica TCS-SP5 (Leica Microsystems, Madrid, Spain) confocal microscope. In Figure 6, region (i) is referred to the nearest 50 µm and region (ii) between 50 and 100 µm. Images were processed with the program ImageJ 1.52e (ImageJ software, National Institutes of Health, USA).

### 2.14. Statistics

**Figure 1:**
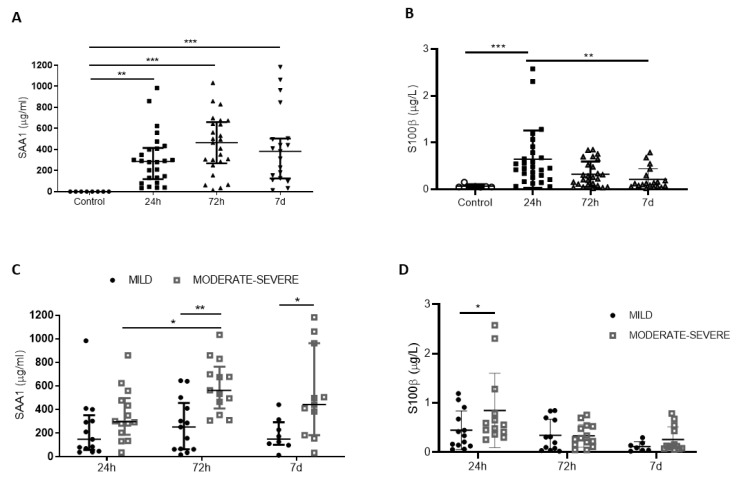
SAA1 levels remains increased the first week after trauma in serum of TBI patients, while S100B just raises at 24 h. (**A**) SAA1 levels increased at 24 h (n = 29), 72 h (n = 28), and 7 days (n = 21) after trauma compared to healthy subjects (controls, n = 8). ** *p* < 0.01, *** *p* < 0.001, Kruskal–Wallis with Dunn’s multiple comparisons test. (**B**) S100B levels significantly incremented at 24 h (n = 29), but not at 72 h (n = 28) and 7 days (n = 21) after trauma. ** *p* < 0.01, *** *p* < 0.001, Kruskal–Wallis with Dunn’s multiple comparisons test. (**C**) Patients were classified in two groups of severity (mild and moderate/severe) according to GCS. SAA1 serum levels correlated with severity at 72 h and 1 week post-trauma. * *p* < 0.05, ** *p* < 0.01, 2-way ANOVA with Sidak’s multiple comparisons test. (**D**) Patients were classified in two groups of severity (mild and moderate/severe) according to GCS. S100B levels correlated with severity at 24 h. * *p* < 0.05, 2-way ANOVA with Sidak’s multiple comparisons test. Data of all experiments are represented as median with interquartile range.

**Figure 2:**
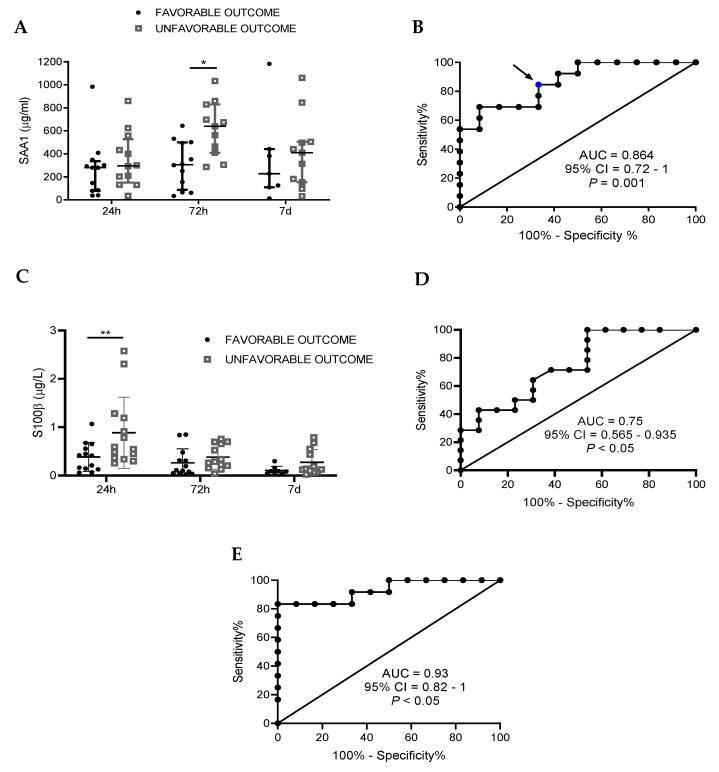
SAA1 levels 72 h after trauma predict TBI patients’ outcome at 6 months, S100B at 24 h, and a combination of both biomarkers improves the ability of prediction. Functional outcome was assessed 6 months after trauma using the GOSE test and patients were divided in two groups: favorable and unfavorable outcome. (**A**) At 72 h, SAA1 levels were significantly higher in the unfavorable outcome group. * *p* < 0.05, 2-way ANOVA with Sidak’s multiple comparisons test. (**B**) Receiver operating curve (ROC) for SAA1 levels at 72 h after hospital admission to predict possible differences between “favorable” (n = 13) and “unfavorable” (n = 13) outcome. Blue dots represent the value for sensitivity and specificity shown in Table 2. (**C**) At 24 h, S100B in serum is increased in the unfavorable outcome group. ** *p* < 0.01, 2-way ANOVA with Sidak’s multiple comparisons test. (**D**) Receiver operating curve (ROC) for S100B levels at 24 h after hospital admission to predict possible differences between “favorable” (n = 13) and “unfavorable” (n = 13) outcome. (**E**) Receiver operating curve (ROC) that combines SAA1 (72 h) and S100B (24 h) values to determine the discriminative capacity of the biomarker combination in outcome 6 months after the injury (favorable, n = 13; unfavorable, n = 13). Data of 2A and 2C are represented as median with interquartile range.

**Figure 3:**
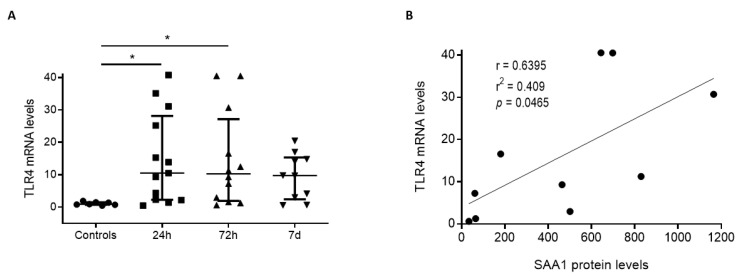
TLR4 mRNA levels increase in circulating leucocytes during the first week after trauma and determination at 72 h positively correlates with SAA1 serum levels. (**A**) Q-PCR determined that TLR4 mRNA levels of circulating blood cells significantly increase at 24 h (n = 13) and 72 h (n = 11) in trauma patients compared to controls (n = 7). At 7 days (n = 10), there were no significant differences. * *p* < 0.05, 1-way ANOVA with Dunnett’s multiple comparisons test. (**B**) A correlation analysis (10 patients) between TLR4 mRNA of blood cells and SAA1 serum levels at 72 h was determined. The correlation coefficient (r = 0.6395) detected a positive correlation between the variables. In the correlation analysis test, *p* < 0.05 was established to determine a true correlation. Data of all experiments are represented as median with interquartile range.

**Figure 4:**
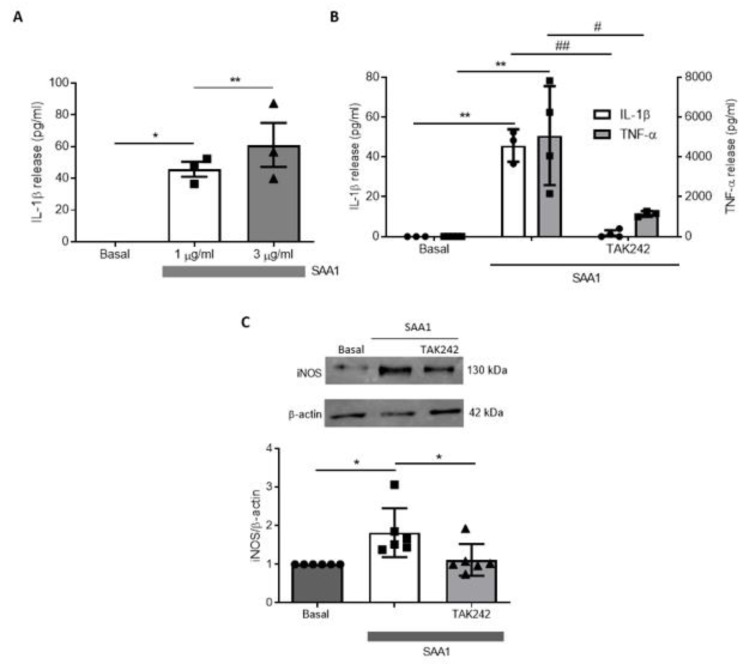
TAK242 treatment prevented the increase of proinflammatory parameters induced by SAA1 in primary mixed glial cultures. (**A**) Glial cultures were treated with SAA1 (1 and 3 µg/mL) with or without TAK242 (1 µM) during 24 h. ELISA revealed a significant increase of IL-1β release to culture medium (n = 3). * *p* < 0.05, ** *p* < 0.01, 1-way ANOVA test with Tukey’s multiple comparisons test. (**B**) ELISA analysis revealed that TAK242 prevented the increase of IL-1β (n = 4) and TNF-α (n = 5) produced by SAA1. * *p* < 0.05, ** *p* < 0.01, # *p* < 0.05, ## *p* < 0.01,1-way ANOVA test with Tukey’s multiple comparisons test. (**C**) Representative immunoblot and quantification of iNOS expression in cultures treated with SAA1 with or without TAK242 (n = 6). * *p* < 0.05, 1-way ANOVA test with Tukey’s multiple comparisons test. Data of all experiments are represented as mean ± SD.

**Figure 5:**
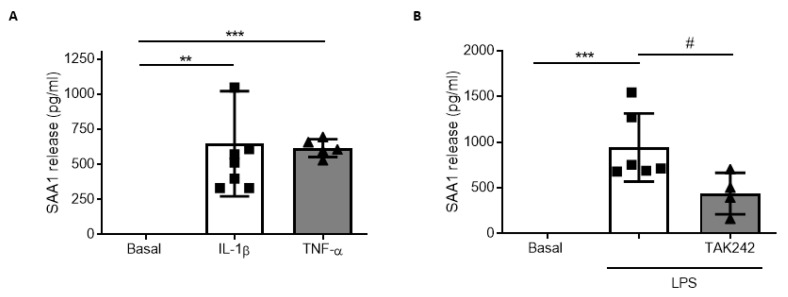
Glial cells synthetized and released SAA1 after inflammatory stimuli. (**A**) Recombinant proteins IL-1β (10 ng/mL) and TNF-α (10 ng/mL) produced the release of SAA1 (detected by ELISA) to the culture medium of mixed glial cultures (n = 6). * *p* < 0.05, ** *p* < 0.01, 2-tailed unpaired t test. (**B**) Primary mixed glial cultures treated with LPS 1 µg/mL stimulated SAA1 release, which was reverted by TAK242 (1 µM) (n = 5). * *p* < 0.05, *** *p* < 0.001, # *p* < 0.05, 1-way ANOVA test with Tukey’s multiple comparisons test. Data of all experiments are represented as mean ± SD.

**Figure 6:**
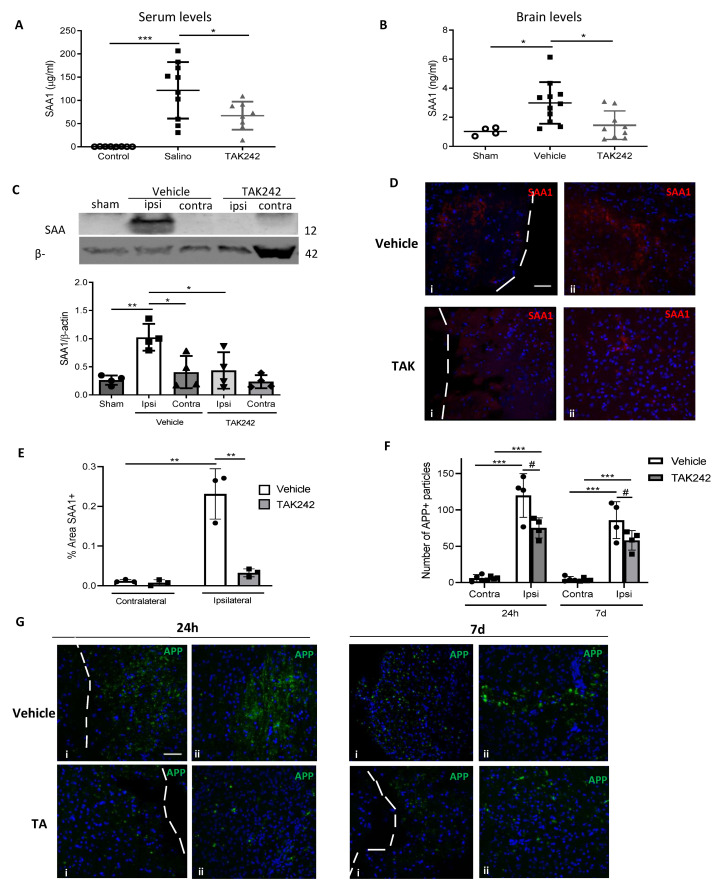
TLR4 antagonism decreased serum and brain SAA1 levels 1 day after TBI. (**A**) TAK242 (3 mg/kg) reduced serum SAA1 protein levels. Mice were treated 1 h after CHI (sham, n = 8; vehicle, n = 10; TAK, n = 8). * *p* < 0.05 and *** *p* < 0.001, 1-way ANOVA with Tukey’s multiple comparisons test. (**B**) Treatment reduced cerebral accumulation of SAA1 at one day post-injury (sham, n = 4; vehicle, n = 11; TAK, n = 9). * *p* < 0.05, 1-way ANOVA with Tukey’s multiple comparisons test. (**C**) Brain lysates of mice treated with or without TAK242 were immunoblotted with SAA1 antibody and protein expression was quantified (sham, n = 4; vehicle ipsi and contra, n = 4; TAK242 ipsi and contra, n = 4). * *p* < 0.05, ** *p* < 0.01, 1-way ANOVA with Tukey’s multiple comparisons test. (**D**) SAA1 immunostainings and (**E**) quantification of SAA1 positive area of (i) and (ii) brain regions of mice subjected to TBI at the indicated time points, treated or not with TAK242 (vehicle ipsi and contra, n = 3; TAK242 ipsi and contra, n = 3). ** *p* < 0.01, 2-way ANOVA with Sidak’s multiple comparisons test. (**F**) Quantification of APP particles of (i) and (ii) regions and (**G**) APP immunostainings of brains of mice subjected to TBI at the indicated time points, treated or not with TAK242 (vehicle ipsi and contra, 24 h and 7 d, n = 4; TAK242 ipsi and contra, 24 h and 7 d, n = 4). *** *p* < 0.01, # *p* < 0.05, 2-way ANOVA with Sidak’s multiple comparisons test. Photos show the damaged hemisphere in an area immediately surrounding the injury site ((i) the nearest 50 µm) and in a deeper area ((ii) between 50 and 100 µm). Images are representative of each group. Scale bars: 5 µm. White arrows indicate the limit of the cortical damaged area. Data of all experiments are represented as mean ± SD. Ipsi, ipsilateral hemisphere; contra, contralateral hemisphere.

**Figure 7:**
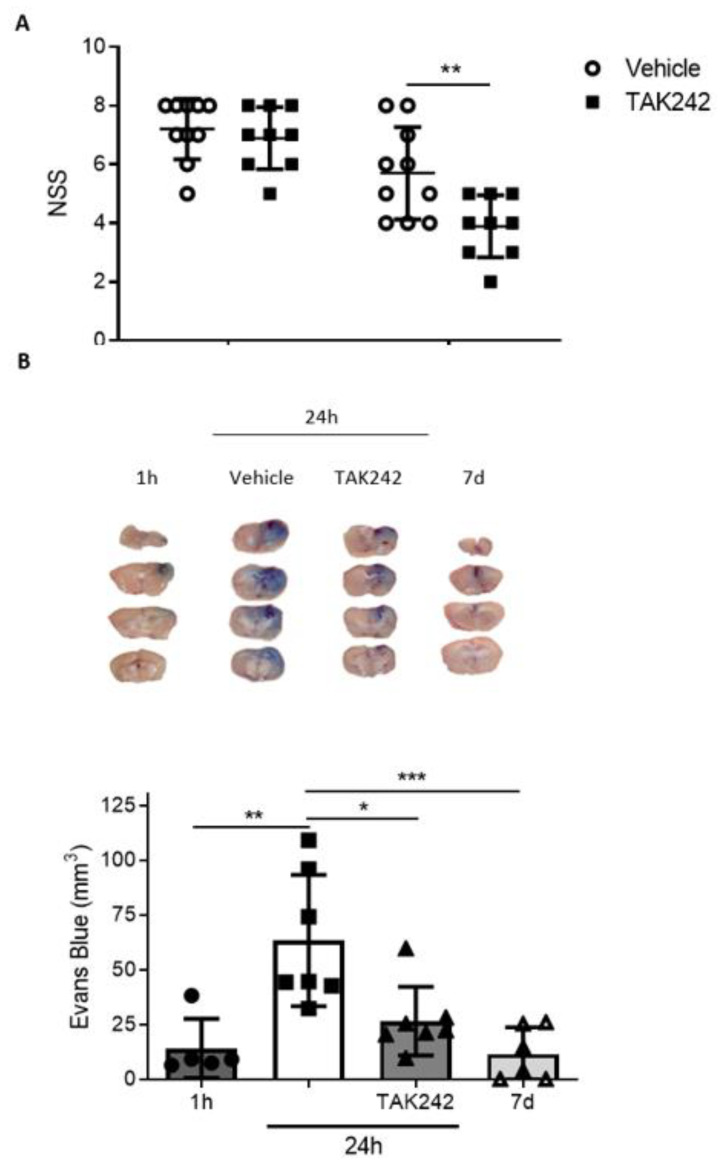
TAK242 improves NSS score and Blood–Brain Barrier (BBB) impairment produced by trauma. (**A**) Immediately following the 1-h NSS test, mice were treated with vehicle solution (0.9% NaCl) or TAK242 (3 mg/kg). NSS was repeated in the same mice 1 d.a.i (vehicle, n = 10; TAK, n = 8). ** *p* < 0.01, 2-way ANOVA with Sidak’s multiple comparisons test. (**B**) Mice were injected intraperitoneally with Evans Blue dye (2%) after the 1-h NSS test in vehicle and TAK242 groups and were sacrificed 24 h later. For 1 h group, it was injected immediately after TBI. For 7 d group, Evans Blue was injected the day before sacrifice. Brains were cut in 2 mm slices to analyze the stained brain surface (1 h, n = 5; vehicle, n = 7; TAK242, n = 7; 7 d, n = 6). * *p* < 0.05, ** *p* < 0.01, *** *p* < 0.001, 1-way ANOVA with Tukey’s multiple comparisons test. Representative photographs of the four 2-mm slices obtained in each brain are shown above the graph.

Data are represented as median with interquartile range (Figure 1 and Figure 2, and Figure S1 or means ± SD (Figure 3, Figure 4, Figure 5, Figure 6 and Figure 7 and Figures S2 and S3). The analysis of normality was assessed by D’Agostino–Pearson omnibus normality test for human and “in vivo” experiments. Data distribution for “in vitro” studies was assumed to be normal. Statistical significance between two groups was measured by 2-tailed unpaired *t*-test. For differences between more than two groups with one independent factor, we used 1-way ANOVA with Tukey’s multiple comparisons test when data followed normal distribution and the nonparametric Kruskal–Wallis test with Dunn’s multiple comparisons test for data that not followed normality. The experiments that had two different independent variables were analyzed by 2-way ANOVA with Sidak’s multiple comparisons test. Animals were randomly assigned to the different groups before TBI induction and NSS evaluation was blinded. *p* < 0.05 was considered statistically significant. All statistical analysis and graphical representation were performed using GraphPad Prism 6.0 software program (GraphPad Software Inc., San Diego, CA, USA. RRID:SCR_002798). 

The discriminatory ability of SAA1/S100B levels at hospital admission regarding dichotomized outcome and TBI detection was evaluated using the area under the receiver operating characteristic curves. Sensitivity, specificity, and positive and negative predictive values were calculated from contingency tables. Optimal cut points estimated from ROC curves were selected prioritizing sensitivity (as “rule-out” test). Logistic regression analysis was used to determine whether SAA1/S100B levels remained as an independent prognostic factor for dichotomized outcome at 6 months when adjusted for other known factors related to outcome. Data were analyzed with statistical package SPSS version 21.0 (IBM Corporation, Armonk, NY, USA). *p* < 0.05 was considered statistically significant.

## 3. Results

### 3.1. Expression Profile of SAA1 and S100B and Their Correlation with Injury Severity 

Table 1 shows the demographic characteristics of TBI patients included in the study. First, we examined the levels of SAA1 protein at different times (24 h, 72 h, and 7 days) after injury (Figure 1). We observed a significant increase in SAA1 serum levels after 24 h (median = 280 µg/mL; IQR = 73.41–404.4), 72 h (median = 381.2 µg/mL; IQR = 161.9–642.9), and 7 days (median = 313.2 µg/mL; IQR = 117.8–500.4) post-TBI compared to control patients (Figure 1A). A peak and a plateau were reached at 72 h, which was maintained significantly elevated at all time points after the injury. Changes in SAA1 levels in peripheral blood did not differ between male and female TBI patients, indicating no sex effects (Figure S1). In the case of S100B, we only observed a significant increase in serum levels at 24 h (median = 0.45 µg/L; IQR = 0.26–0.78), while values at 72 h (median = 0.25 µg/L; IQR = 0.09–0.55) and 7 days (median = 0.12 µg/L; IQR = 0.06–0.29) were progressively decreasing (Figure 1B). 

Next, we determined the correlation between SAA1 and S100B levels with clinical parameters. First, we classified patients according to Glasgow Coma Scale (GCS) into two groups: moderate–severe TBI (moderate, GCS 9–13; severe, GCS 3–8) and mild TBI (GCS 14–15) (Table 1). Figure 1C shows no significant changes in SAA1 levels among the mild-TBI group at any time tested. However, SAA1 levels in the moderate–severe group were significantly higher at 72 h in comparison to 24 h, confirming that maximum SAA1 concentrations were reached 3 days after the injury. Furthermore, we found significant differences between mild and moderate–severe groups at 72 h and 1 week after TBI. Regarding S100B, only the values at 24 h were able to discriminate injury severity of patients (Figure 1D). These data suggest SAA1 as an important mediator of the inflammatory response in TBI, as it participates in the APR and remains altered in systemic circulation during the first week post-injury.

### 3.2. Correlation of SAA1 Alone or in Combination with S100B with Neurological Outcome 6 Months after TBI 

To determine the neurological outcome of TBI patients 6 months after TBI, we used the Extended Glasgow Outcome Scale (GOSE), a global test to determine long-term functional outcome of each patient after trauma [18]. Thus, we dichotomized patients into two groups: unfavorable (GOSE 1–5) and favorable (GOSE 6–8) outcome. SAA1 remains stable in patients with a favorable outcome, while plasma protein concentrations in the unfavorable outcome group increased at 72 h after the injury (Figure 2A). This elevation was significantly higher in the unfavorable compared with favorable outcome. To evaluate whether serum SAA1 levels 72 h after TBI could discriminate between patients with favorable and unfavorable outcomes, a ROC curve was calculated (Figure 2B). The area under the curve (AUC) verified that SAA1 levels 72 h after TBI could discriminate between the favorable and unfavorable outcomes of patients (AUC = 0.86, 95% CI = 0.72–1, *p* = 0.001). With sensitivity set at 100%, SAA1 displayed a specificity of 46.2%. We also evaluated SAA1 in the best scenario setting sensitivity in the 90 to 100% range. In this case, SAA1 reached 53.8% specificity, lowering its sensitivity to 92.3%. Moreover, we chose the cut-off level of 381.2 µg/mL (blue dot in Figure 2B) as the best value to discriminate between favorable and unfavorable outcome 6 months after the injury. We obtained a sensitivity of 84.62% and specificity of 69.2%. Finally, we calculated the positive and negative predictive values (PPV and NPV) at this cut-off level, and the obtained values were 73.3% and 80%, respectively (Table 2).

**Table 2:** Clinical performance characteristics of SAA1 levels at 72 h as a predictor of outcome. Sensitivity, specificity, PPV and NPV for the cut-off point selected are showed. PPV = Positive Predictive Value; NPV = Negative Predictive Value.

Cut-Off (µg/mL)	Sensitivity (%)	Specificity (%)	PPV (%)	NPV (%)
>381.2	84.62	69.2	73.3	80

Regarding S100B, Figure 2C shows that protein levels measured at 24 h were significantly higher in the unfavorable group. Moreover, the ROC curve demonstrated that S100B levels measured at 24 h predicts patients’ outcome at 6 months (Figure 2D) and, as in the case of SAA1, it could discriminate between favorable and unfavorable outcomes of patients (AUC = 0.75, 95% CI = 0.565–0.935, *p* < 0.05). Finally, in order to try to enhance prognosis capacity, we combined both biomarkers in a ROC curve (Figure 2E). As a result, we obtained higher AUC values with the combination of both biomarkers than isolated ones (AUC = 0.93, 95% CI = 0.8267–1, *p* < 0.001). To sum up, we found that SAA1 discriminates patients according to the severity of TBI and its measurement 3 days post-injury predicts the outcome of the patients 6 months later. Furthermore, the combination of SAA1 and S100B measurements improved the prognosis of outcome compared to each biomarker analyzed independently.

### 3.3. Positive Correlation between SAA1 Serum Levels and TLR4 mRNA in White Blood Cells from Human TBI Patients

Endogenous SAA1 signals via TLR2/4 in different immune cells [19,20,21,22,23]. On the other hand, TLR2/4 expression is associated to outcome in stroke patients [24]. To examine whether SAA1 is relevant for inflammation after TBI, we studied TLR4 expression on white blood cells (WBC) of TBI patients 24 h, 72 h, and 7 days after TBI. As in the case of SAA1, we observed an increase in TLR4 mRNA at 24 h (median = 10.51; IQR = 2.26–28.15), 72 h (median = 11.24; IQR = 2.42–15.34), and 7 days (median = 9.74; IQR = 2.42–15.34) post-TBI (Figure 3A). Significant differences were obtained at 24 h and 72 h after TBI. Next, we examined the relationship between TLR4 expression and serum SAA1 levels. A positive correlation was found at 72 h post-TBI (Pearson coefficient = 0.6395, *p* < 0.05) (Figure 3B). Altogether, our data indicate that serum SAA1 levels, TLR4 expression, and the severity and the outcome of patients could be mechanistically linked.

### 3.4. SAA1 Contributes to Inflammation in Glial Cultures via TLR4 Activation

Once a positive correlation between SAA1 serum levels and TLR4 mRNA with significant predictive value for severity and patient outcome was established, we explored the role of SAA1 in brain inflammation. For this purpose, we used primary mixed glial cultures from mice treated with SAA1 recombinant protein at 1 µg/mL and 3 µg/mL and determined the levels of the proinflammatory cytokine IL-1β (Figure 4A). At both concentrations tested, we obtained a significant increase in IL-1β release levels, so we selected the concentration of 1 µg/mL to perform further experiments. After 24 h of incubation, we detected an increase in the proinflammatory cytokines TNF-α and IL-1β by ELISA (Figure 4B), and an increase in the inducible nitric oxide synthase (iNOS) enzyme by Western blot (Figure 4C). To further demonstrate that SAA1 exerts its inflammatory response through TLR4 receptors [25,26], we co-treated glial cells with SAA1 1 µg/mL with TAK242 (1 µM), a specific TLR4 antagonist, for 24 h. We obtained a significant reduction in the release of TNF-α and IL-1β to the culture medium (Figure 4B), as well as in iNOS protein levels in cell lysates (Figure 4C).

Once proven that SAA1 can trigger an inflammatory response in glial cells, we examined whether proinflammatory stimuli such as IL-1β and TNF-α (10 ng/mL) enhanced SAA1 synthesis and release. SAA1 levels in glial cultures were significantly increased by IL-1β and TNF-α (Figure 5A). We then treated cultured glia with LPS (1 µg/mL), a well-known TLR4 agonist, to trigger inflammatory cascade. Twenty-four hours of LPS treatment induced an increase in the levels of SAA1 that was reduced by ~50% in the presence of TAK242 (Figure 5B). These results indicate that SAA1 generates a positive feedback loop in glial cells. First, SAA1 and other DAMPs promote the release of inflammatory cytokines (IL-1β and TNF-α) that in turn stimulate SAA1 production, which potentiate the inflammatory response. TLR4 antagonism decreases SAA1 release, thereby reducing the effects of this loop.

### 3.5. Post-TBI Treatment with the TLR4 Antagonist TAK242 Reduces SAA1 Levels

Having verified that TAK242 reduced the downstream effects of SAA1 in vitro, we tested if the compound had beneficial effects in vivo. C57BL/6J mice subjected to CHI were treated for 1 h post-TBI with TAK242 (3 mg/kg, i.p.). First, we assessed SAA1 levels in blood serum and brain parenchyma 24 h after the injury. We observed that TLR4 antagonism significantly reduced SAA1 levels compared to non-treated animals in both serum and brain (Figure 6A,B). No differences were detected neither in serum nor cerebral cortex 1 week after TBI between vehicle and TBI groups (Figure S2). To confirm these findings, we performed Western blot and immunofluorescence analysis against SAA1. Immunoblotting of cerebral tissue revealed that SAA1 is not present in brains of non-injured animals (Figure 6C). The levels of SAA1 increased in the ipsilateral hemisphere of TBI mice, while they were undetectable in the contralateral hemisphere. Moreover, treatment of TBI animals with TAK242 diminished SAA1 accumulation in brain parenchyma of the injured hemisphere (Figure 6C). Next, we performed immunofluorescence of SAA1 in brain slices from TBI animals (Figure S3A shows the analyzed regions). SAA1 labeling was detected 24 h post-injury in the damaged area, as well as in the penumbra area of the ipsilateral hemisphere (Figure 6D). TAK242 treatment reduces SAA1 staining in the damaged region (Figure 6D). Quantification of the SAA1-positive area confirmed that TAK242 almost completely reduced brain levels of the protein (Figure 6E). No SAA1 staining was detected in the contralateral hemisphere at 24 h or in the ipsilateral and contralateral areas 7 days after TBI (Figure S3B–D). These findings suggest that SAA1 is an important mediator of the acute inflammatory response, as SAA1 levels arise in systemic circulation and, most important, in brain parenchyma 24 h after TBI in mice.

Amyloid precursor protein (APP) is used as an indicative of axonal injury as it accumulates as a result of axonal transport system disruption [27]. Therefore, to confirm the presence of axonal injury in our model, we used immunofluorescence of APP in brain slices from TBI animals. We found APP presence at both 24 h and 7 d.a.i in the ipsilateral cortex (Figure 6G), but not in contralateral cortex (Figure S3B). We also evaluated the possibility that TAK242 could prevent damage spreading along the injury site. APP particles were significantly lowered 24 h and 7 d.a.i by the treatment with TAK242 (Figure 6F,G), which means that TLR4 antagonism reduces the extent of diffuse axonal injury both at 24 h and 7 days after TBI.

### 3.6. Treatment with TAK242 Improves Neurobehavioral Outcome and Prevents Blood-Brain Barrier Disruption 

As TLR4 is one of the main contributors to inflammation in brain [9,10,28], we explored the possible effects of its inhibition in the functional outcome of animals subjected to CHI. We used the neurological severity score (NSS) test, which evaluates neurologic, motor, and behavioral skills in mice after TBI [17]. A score of 10 points represents a total neurological impairment, whereas 0 points means a normal function. One hour after TBI, we detected that animals obtained a NSS between 6 and 8 (sham animals had a score of 0 or 1, data not shown). Following the 1-h test, animals were treated with TAK242 (3 mg/kg) and NSS was assessed again 24 h after the injury. In this case, we found that TAK242-treated animals significantly improved neurobehavioral skills compared to saline vehicle animals (Figure 7A). Next, we examined if the NSS improvement was related to a reduction in BBB breakdown. For that, we measured the extravasation of Evans Blue dye into cerebral tissue at different time points after CHI. First, we analyzed Evans Blue leakage in non-injured mice, and we did not find stained areas (data not shown). One hour post-TBI, Evans Blue dye only penetrated in the cortex surrounding the injury, while the following day, vehicle-treated mice had almost the whole ipsilateral hemisphere stained (Figure 7B). TAK242 treatment 1 h after TBI significantly reduced Evans Blue leakage in brain parenchyma compared to those vehicle-treated group (Figure 7B). Finally, we assessed the BBB permeability 7 d.a.i. and found a spontaneous recovery of vasculature stability in our model of TBI (Figure 7B). This evidence reveals that TLR4 plays an important role in BBB disruption and its blockade promotes beneficial effects on neurological parameters after TBI in mice.

## 4. Discussion

Herein, we identified a direct link between SAA1 and TLR4 in traumatic brain injury, which positively correlated with injury severity and 6-month outcome of patients, as well as with BBB breakdown and worse outcome in animals. The use of plasma biomarkers is a priority in current personalized medicine. In the context of TBI, biomarkers have the potential to be used as diagnostic markers of injury severity, markers of response to treatment (monitoring biomarkers), or even as predictors of outcomes after trauma (predictive biomarkers). As most biomarker validations are conducted 24 h after brain trauma [29,30,31], the time course of biomarkers has been poorly studied. However, some groups have recently started to determine the kinetics of TBI biomarkers at longer times to predict outcome and other clinical parameters [32]. We provide here an example of a time course evaluation of a potential biomarker combination for prediction and, possibly, for patient follow-up and monitoring drug responses. The predictive value of serum SAA1 is better at 72 h than 24 h or 7 days after brain trauma. Moreover, it improves the predictive value obtained for S100B at 24 h after TBI. However, the most important point is that the combination of both biomarkers (measured at different times post-TBI, 24 h for S100B and 72 h for SAA1) has the best predictive value of all combinations tested. On the other hand, we have observed a possible mechanistic link between inflammation and outcome of TBI patients, as values of serum SAA1 positively correlates with TLR4 mRNA obtained from WBC from patients at the same time point (72 h). Recently, it has been shown that TLR4 expression in WBC correlates with poor outcome in ischemic stroke patients [24,33]. Therefore, as in stroke, an important inflammatory response is triggered after TBI that could be dramatically increased by the important rise of SAA1 serum levels.

Clinically, it is important to understand the pathophysiological alteration of SAA1 in TBI patients. Our data suggest that SAA1 remained elevated up to 1 week post-TBI. Considering the results obtained in in vitro and in vivo models, it is plausible that SAA1 activates the TLR4 of inflammatory cells inducing the release of inflammatory mediators such as interleukins (IL-1β, TNF-α, among others). These inflammatory mediators, in turn, increase SAA1 through activation of their respective inflammatory receptors. We propose that this cascade of events produces a positive inflammatory loop that serves to perpetuate the inflammatory response. Thus, blocking one step of this inflammatory loop may represent a suitable therapeutically option in TBI patients. This is the case of the TLR4 blocker resatorvid (TAK242), which improves the outcome of animals, avoids BBB breakdown, and reduces the brain levels of SAA1.

The role of inflammation in the progression of different diseases, like neurodegenerative diseases, is well known [34,35,36]. We have shown that recombinant SAA1 activates TLR4 inflammatory receptor in vitro. Our data are in line with the literature, describing the participation of SAA1 in the inflammatory response of TLR2/4 activation in periodontitis [23], myotube atrophy [37], or sepsis [20], among others. Our results may help to shed light about the relationship between SAA1-TLR4 axis and BBB breakdown. Thus, TLR4 blockade could improve BBB breakdown, and thereby reduce to control levels the presence of SAA1 in the brain. It has been recently described that overexpressed liver-derived SAA1 protein in mice accumulates in the brain crossing the BBB, triggering a depressive-like behavior of mice [38]. Moreover, in double transgenic APP/SAA1 mice, SAA1 exacerbated amyloid aggregation, glial activation, causing greater memory decline in APP/SAA1 compared to single transgenic APP mice [39]. Other studies in humans showed that SAA1 was detected in senile plaques in Alzheimer’s disease tissue, predominantly localized to neuritic plaques [40]. All together, these studies point to brain SAA1 levels as a trigger of amyloid aggregation or co-aggregation with SAA1. This pathological process may be taking place in chronic traumatic encephalopathy (CTE) of TBI patients in collision sports, which may lead to worsen the cognitive function of TBI patients.

Of clinical importance, our data suggest a mechanistic biomarker-guided stratification, using SAA1 protein and TLR4 mRNA. Therefore, we would modulate drug treatment to those patients with an unfavorable outcome, which have a high likelihood to worsen from this inflammatory loop, and thus reduce the number of patients with poor prognosis. In addition, focusing on biomarkers that share the SAA1-TLR4 inflammatory axis will ensure that a personalized and not standard dose can be applied and will further reduce possible dropouts. Finally, our findings provide a clear rationale for further development of a pharmacological SAA1/TLR4 axis inhibition as a first-in-class pharmacological strategy to stop unfavorable outcome in TBI patients.

## 5. Patents

Víctor Farré-Alins, Alejandra Palomino-Antolín, Paloma Narros-Fernández, Juliana M. Rosa, José María Roda-Frade, Cristina S. Carabias, Santiago Yus-Teruel, Luis Gandía, Alfonso Lagares, Borja J. Hernández-García and Javier Egea. Método para determinar la evolución del daño cerebral agudo y composición farmaceútica para su tratamiento. EP202031194.

## Figures and Tables

**Table 1:** Demographic details of the patient cohort.

	Controls	24 h	72 h	7 d
N (%) *	8	29 (85.3)	28 (82)	21 (61.7)
Sex, male/female	4/4	22/7	21/7	16/5
Age, mean	44 ± 17.6	52.8 ± 18.3		
Severity (GCS)				
mild		15	15	10
moderate		4	3	4
severe		10	10	7
Outcome at 6 months (GOSE)				
Favorable		13	13	8
Unfavorable		13	13	11

* Total number of patients was 34. It was not possible to obtain all the samples at the three time points from the whole cohort. Some patients were lost in the GOSE as it was impossible to follow the patients 6 months after the injury.

## Supplementary Materials

Supplementary materials can be found at https://www.mdpi.com/article/10.3390/biomedicines9060599/s1. Figure S1. Gender did not affect to SAA1 concentrations after trauma. There were no statistical differences between men and women in SAA1 levels at 24 h, 72 h and 1 week post-injury. 2-way ANOVA with Sidak’s multiple comparisons test. Data are represented as median with interquartile range. Figure S2. SAA1 levels increased in serum and brain parenchyma 24 hours after traumatic brain injury and returned to basal levels 7 days post-injury. (A) ELISA of SAA1 determined an early increase in protein levels that diminished 7 d.a.i. (sham, n = 8; 1 d, n = 10; 7 d, n = 8). *** *p* < 0.001, 1-way ANOVA with Tukey’s multiple comparisons test. (B) TBI lead to SAA1 accumulation in the injured hemisphere 1 d.a.i., which decreases 1 week later (sham ipsilateral and contralateral, n = 4; 1d ipsilateral and contralateral, n = 11; 7d ipsilateral and contralateral, n = 5). * *p* < 0.05, ** *p* < 0.01, 2-way ANOVA with Sidak’s multiple comparisons test. Data of all experiments are represented as mean ± SD. Figure S3. SAA1 returns to basal levels and APP remains expressed 7 days after injury in the ipsilateral cortex. (A) Illustration showing the areas where immunofluorescence images were taken in the ipsilateral hemisphere. (B) Coronal sections of brains of mice subjected to TBI were immunostained for SAA1 and APP at 24 h or 7 days after trauma. DAPI was used to stain nucleus. Microscopy pictures were taken at the ipsilateral hemisphere, in an area immediately surrounding the injury site (i, the nearest 50 µm) and in the adjacent area (ii, between 50 and 100 µm). (C) Illustration showing the areas where immunofluorescence images were taken in the contralateral hemisphere. (D) Immunofluorescence images for SAA1 and APP in the contralateral hemisphere of injured mice brains 24 h or 7 days post-TBI. Scale bars: 5 µm. White arrows indicate the limit of the cortical damaged area.
