# Supplementary material for: Serum Amyloid A1/Toll-Like Receptor-4 Axis, an Important Link between Inflammation and Outcome of TBI Patients
Source: Biomedicines. 2021 May 25;9(6):599. doi: 10.3390/biomedicines9060599 (PMC8227125; doi:10.3390/biomedicines9060599)
Supplement: Supplementary file 1 [file biomedicines-09-00599-s001.zip › biomedicines-1211582-supplementary.pdf]

6. Supplemental Figures

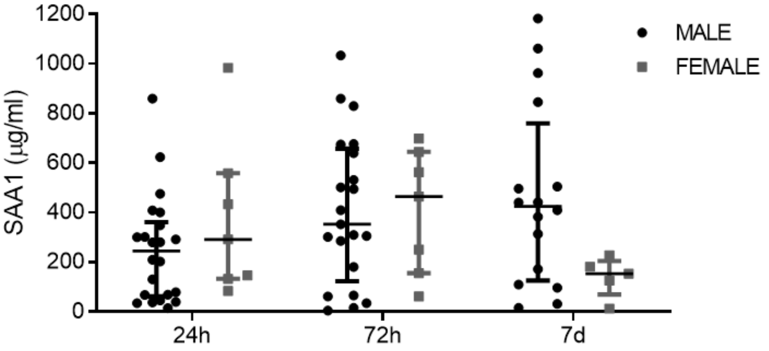

Supplemental Figure 1. Gender did not affect to SAA1 concentrations after trauma. There were no statistical differences between men and women in SAA1 levels at 24h, 72h and 1 week post-injury. 2-way ANOVA with Sidak's multiple comparisons test. Data are represented as median with interquartile range.

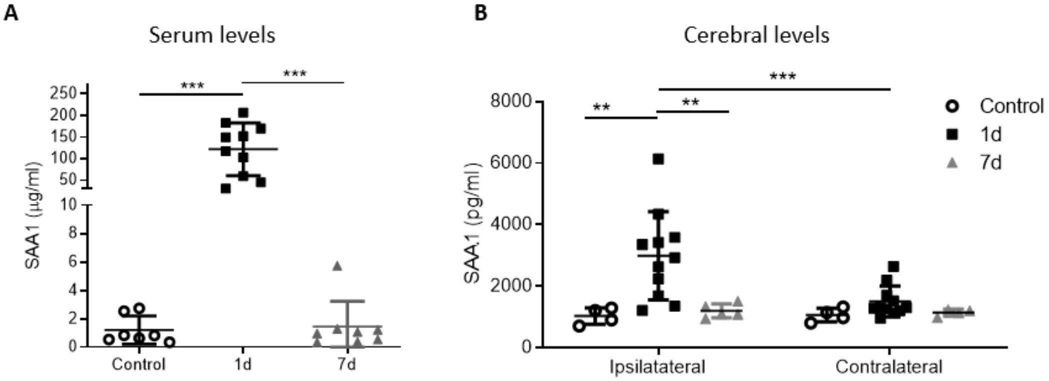

Supplemental Figure 2. SAA1 levels increased in serum and brain parenchyma 24 hours after traumatic brain injury and returned to basal levels 7 days post-injury. (A) ELISA of SAA1 determined an early increase in protein levels that diminished 7 d.a.i. (sham, n = 8; 1d, n = 10; 7d, n = 8). ***P < 0.001, 1-way ANOVA with Tukey's multiple comparisons test. (B) TBI lead to SAA1 accumulation in the injured hemisphere 1 d.a.i., which decreases 1 week later (sham ipsilateral and contralateral, n = 4; 1d ipsilateral and contralateral, n = 11; 7d ipsilateral and contralateral, n = 5). *P < 0.05, **P < 0.01, 2-way ANOVA with Sidak's multiple comparisons test. Data of all experiments are represented as mean ± SD.

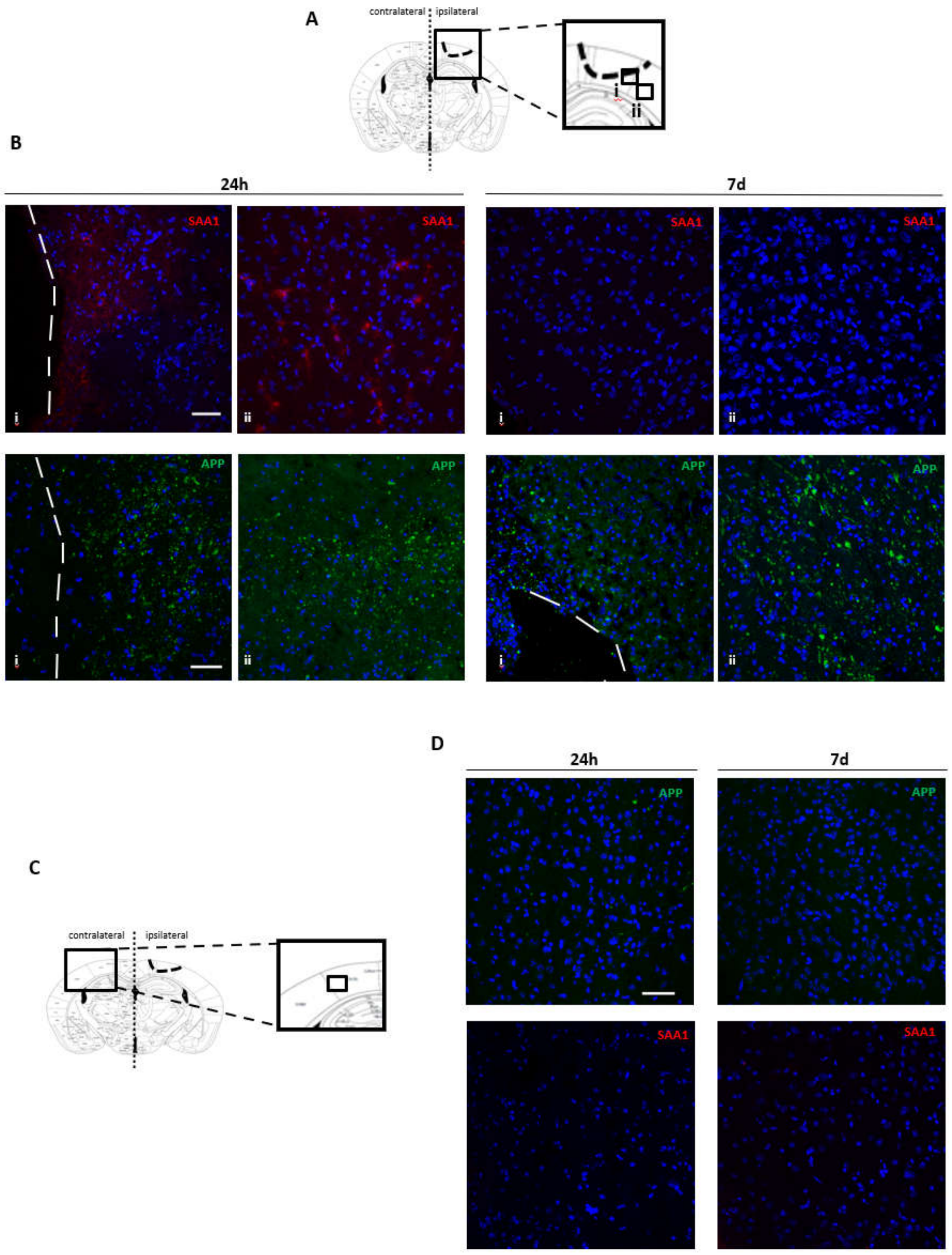

Supplemental Figure 3. SAA1 returns to basal levels and APP remains expressed 7 days after injury in the ipsilateral cortex. (A) Illustration showing the areas where immunofluorescence images were taken in the ipsilateral hemisphere. (B) Coronal sections of brains of mice subjected to TBI were immunostained for SAA1 and APP at 24 h or 7 days after trauma. DAPI was used to stain nucleus. Microscopy pictures were taken at the ipsilateral hemisphere, in an area immediately surrounding the injury site (i, the nearest 50 μm) and in the adjacent area (ii, between 50 and 100 μm). (C) Illustration showing the areas where immunofluorescence images were taken in the contralateral hemisphere. (D) Immunofluorescence images for SAA1 and APP in the contralateral hemisphere of injured mice brains 24 h or 7 days post-TBI. Scale bars: 5 μm. White arrows indicate the limit of the cortical damaged area.
